# Reducing Benign Breast Biopsies in Women Aged 35 Years or Younger Using a Clinician–AI–Age Triage Strategy

**DOI:** 10.3390/diagnostics16213491

**Published:** 2026-10-25

**Authors:** Nisha Sharma, Jack Baldwin, Stuart Hendry

**Affiliations:** 1 Leeds Teaching Hospitals NHS Trust, Leeds LS9 7TF, UK; jack.baldwin@nhs.net; 2 HecoAnalytics Ltd., Uxbridge UB8 3PQ, UK; stuart@hecoanalytics.com

**Keywords:** breast ultrasound, artificial intelligence, clinical decision support, biopsy avoidance, fibroadenoma, diagnostic accuracy, budget impact, NHS

## Abstract

**Background/Objectives:** Symptomatic breast services face increasing diagnostic demand, while younger women with benign-appearing ultrasound lesions frequently undergo biopsy despite a low prevalence of malignancy. This study evaluated whether a rule-based clinical decision algorithm combining patient age, clinician ultrasound assessment and artificial intelligence (AI) decision support could identify a subgroup in whom biopsy might be avoided without excluding malignancy from the biopsy pathway. **Methods:** A retrospective analysis was performed using prospectively collected service-evaluation data from 240 patients (254 lesions) attending a large NHS breast unit between May and December 2025. Clinician ultrasound assessment, KOIOS DS Breast AI output and histopathology were recorded. Lesions in women aged ≤ 35 years were eligible for biopsy avoidance when clinician assessment was U2 or U3 and the KOIOS AI assessment was BI-RADS 2. Histopathology was the reference standard. Secondary analyses compared clinician and AI diagnostic performance. An illustrative five-year budget impact analysis was undertaken from the NHS provider perspective. **Results:** Among 151 lesions in women aged ≤ 35 years, 146 were benign and five were malignant. The algorithm would have classified 87 lesions for biopsy avoidance (57.6%; 95% CI, 49.3–65.6%). Of these, 67 (77.0%) had a clinician U3 classification. All 87 were benign on histopathology (100%; 95% CI, 95.8–100%), while all five malignant lesions remained in the biopsy pathway. Clinician assessment showed higher specificity than AI (99.1% vs. 75.7%), whereas AI showed higher sensitivity (96.9% vs. 81.3%). Avoidance of 87 biopsies was associated with approximately £85,000 in gross cost offsets during the evaluation period in the illustrative economic model. **Conclusions:** The AI-supported clinical decision algorithm could substantially reduce benign biopsy activity in younger women while maintaining cancer detection in this cohort. The small number of malignancies, single-centre design and biopsy population mean that prospective multicentre validation is required before routine clinical implementation.

## 1. Introduction

NHS symptomatic breast services are managing sustained growth in referrals while being expected to provide rapid diagnostic resolution. National reports have documented substantial increases in breast-clinic activity, and the NHS Faster Diagnosis Standard requires a diagnosis of cancer, or definitive exclusion of cancer, within 28 days of referral [1,2]. Reducing low-value diagnostic procedures without compromising cancer detection is therefore clinically and operationally important.

In younger women, most solid breast lesions are benign. The 2025 RCR guidance has raised the threshold from under 25 years to women under 30 years where biopsy can be avoided when strict imaging criteria for a benign fibroadenoma are met, and some centres have adopted higher age thresholds after local audit. Recent national survey data suggest increasing acceptance of non-biopsy pathways in carefully selected younger women, while studies using adjunctive techniques such as shear-wave elastography have also reported safe biopsy reduction in selected populations [3,4,5,6]. Nevertheless, increasing the age threshold on the basis of age and conventional ultrasound alone can be difficult to implement consistently because the clinical consequence of a missed malignancy is substantial.

Artificial intelligence has the potential to provide an additional, independent layer of risk stratification. In breast imaging, AI systems have shown diagnostic performance comparable with radiologists in some settings, but the clinical value of AI may be greatest when it complements rather than replaces human interpretation. Decision-referral and AI-assisted ultrasound studies suggest that combining clinician judgement with algorithmic assessment can reduce false-positive recommendations while preserving sensitivity [7,8,9,10].

KOIOS DS Breast (KOIOS Medical, Inc., Chicago, IL, USA) is a regulatory-approved, commercially available AI clinical decision-support system for breast ultrasound. In a multicentre reader study, Mango et al. found that KOIOS decision support increased mean reader AUC from 0.83 to 0.87 and improved agreement between readers [11]. Browne et al. reported potential reductions in benign biopsies in a retrospective biopsy cohort but also found that some cancers would have been missed if biopsy decisions had relied on KOIOS alone [12]. However, there is limited evidence on the use of AI within an age- and clinician-dependent biopsy triage rule embedded in an NHS symptomatic breast pathway, or on the potential provider-level resource consequences of such a strategy.

The primary objective of this service evaluation was, therefore, to estimate the proportion of biopsies that could potentially be avoided in women aged ≤ 35 years using an AI-supported rule-based clinical decision algorithm and to assess the observed safety of the no-biopsy classification against histopathology. Secondary objectives were to compare clinician and AI diagnostic performance within the cohort and to explore the potential budget impact of pathway implementation.

## 2. Materials and Methods

### 2.1. Study Design and Governance

This was a retrospective analysis of prospectively collected data from a service evaluation conducted within the Breast Unit at Leeds Teaching Hospitals NHS Trust (LTHT), United Kingdom. The LTHT Data Access committee gave approval for the use of the anonymised data in this study as this was a prospective service evaluation. The analysis used anonymised data collected during routine clinical care, including data from patients aged under 18 years. Consent was waived for this service evaluation, in which the AI score was generated downstream and did not affect standard care or the clinical decision to biopsy. The reporting of this manuscript was informed by the Checklist for Artificial Intelligence in Medical Imaging (CLAIM) 2024 update and the STARD-AI reporting guideline for diagnostic accuracy studies using artificial intelligence [13,14].

### 2.2. Study Population

A total of 240 patients attending the LTHT Breast Unit between May and December 2025 were included, yielding 254 breast lesions for analysis. Fourteen patients had bilateral lesions. Patient age ranged from 14 to 45 years (mean, 32.4 years). Of the 240 patients, 142 (59.2%) were aged ≤ 35 years and 98 (40.8%) were older than 35 years. Nine patients in the ≤35-year group and five patients in the >35-year group had bilateral lesions, corresponding to 151 and 103 lesions, respectively.

The evaluation was conducted at a large regional breast centre in Leeds, a city with an ethnically diverse population and substantial socioeconomic variation [15,16]. Demographic information beyond age was not available in the anonymised analysis dataset; therefore, the ethnic and socioeconomic composition of the study cohort could not be characterised.

All eligible patients undergoing biopsy in the one-stop clinic during the recruitment period were included consecutively, including those aged under 25 years. Lesions that were not biopsied under departmental policy, including U2 lesions managed without biopsy in women aged under 25 years, were not eligible. Data on these non-biopsied lesions were not collected. The current policy within the breast department is to biopsy all solid lesions if women are 25 years or older. If under 25, ultrasound biopsy was performed for lesions classified as BI-RADS 4 or U3. A lesion measuring 3 cm or more would have a biopsy even if classified as U2 or BI-RADS 2. All women with known hereditary risk are managed via the very high risk screening programme and women with moderate family history through a surveillance pathway which is separate to the one-stop clinic. Family history was not documented in the evaluation dataset.

All 254 lesions included in the analysis underwent biopsy, and final histopathological outcomes provided the reference standard for benign or malignant classification. Biopsy results were recorded using the B1–B5 classification, with repeat sampling undertaken where clinically required, including following non-diagnostic B1 results. Biopsy avoidance was assessed retrospectively; no biopsies were withheld on the basis of the proposed algorithm.

### 2.3. Ultrasound Assessment and Reference Standard

Ultrasound examinations were performed within the one-stop symptomatic breast clinic as part of routine diagnostic assessment. Image acquisition and real-time interpretation used the UK U1–U5 ultrasound classification system. Examinations were undertaken by appropriately trained practitioners in accordance with departmental policy. The individual operator was not recorded for this analysis, and results were pooled across readers. The axilla would only be scanned if the operator thought the lesion was suspicious for malignancy. This would not affect the U score as the U score is for the breast lesion. The departmental policy is to perform quadrant scanning of the symptomatic area. During the evaluation, departmental policy followed the 2019 RCR guidance, with an age threshold of under 25 years for biopsy avoidance in presumed fibroadenomas meeting the specified benign imaging criteria [6].

### 2.4. Artificial Intelligence Decision Support

**Figure 1:**
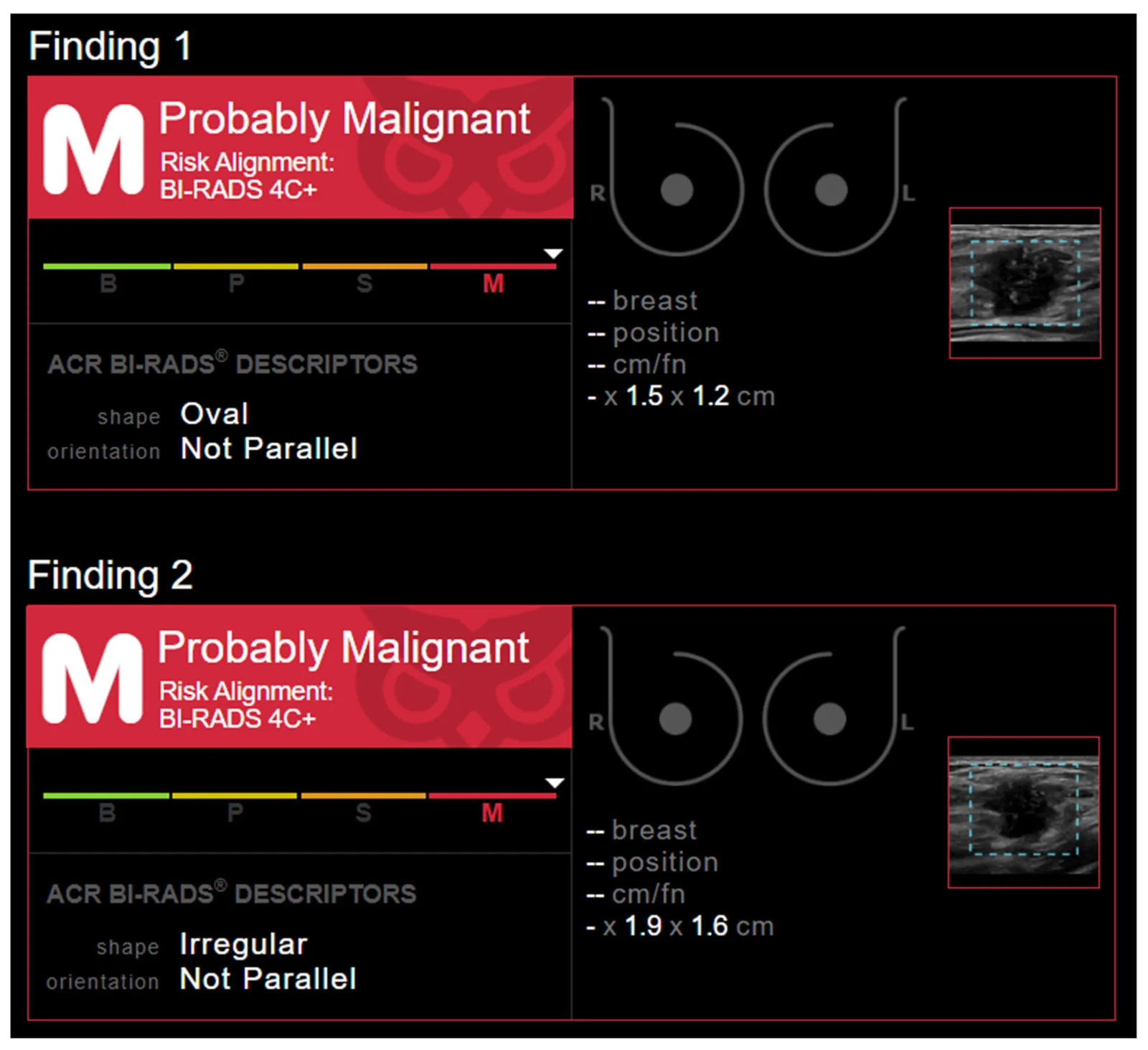
Screenshot from KOIOS software classifying a proven malignant lesion. The red ‘M’ denotes ‘Probably Malignant’ (BI-RADS 4C+); the dashed blue boxes mark the regions of interest.

Ultrasound images were analysed on the PACS workstation using KOIOS DS™ Breast, version 3.6 (KOIOS Medical, Inc., Chicago, IL, USA), which provided lesion classification of BI-RADS 1–4C+, see Figure 1 for example screen capture.

KOIOS was applied to static US images. Ideally we asked for longitudinal and transverse images of the solid lesion. BI-RADS 2 is per the BI-RADS US classification, where likelihood of malignancy is almost 0%. The AI output was performed downstream and without knowledge of the histology or US classification. The region of interest (ROI) covered the outline of the lesion. The ROI was defined by a radiologist with 18 years of experience.

### 2.5. Rule-Based Clinical Decision Algorithm and Outcomes

**Figure 2:**
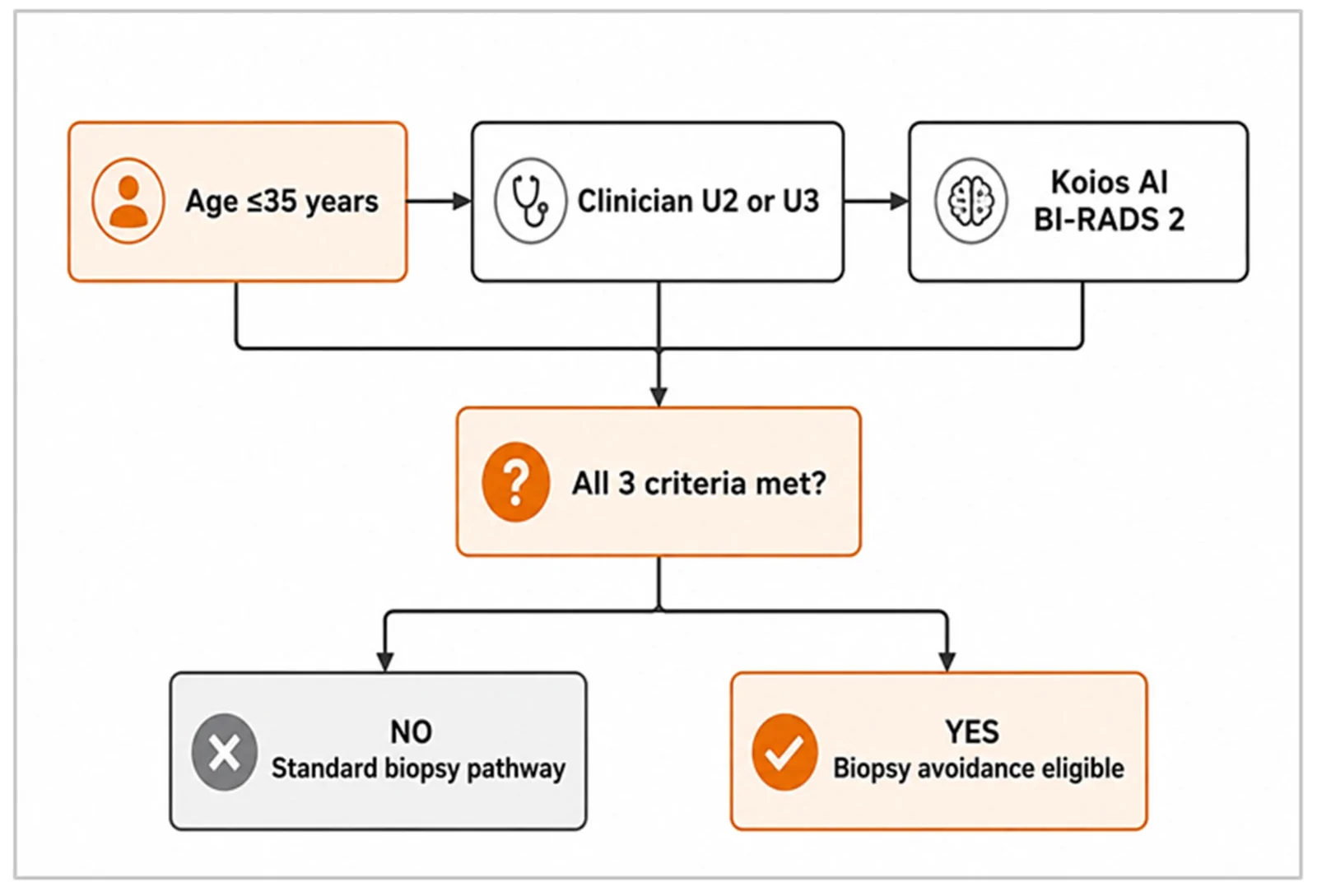
AI-supported rule-based clinical decision algorithm evaluated in the service evaluation. Biopsy avoidance required all three criteria to be met; all other lesions remained in the standard biopsy pathway.

The AI-supported clinical decision algorithm was designed to identify a subset of lesions in younger women for which biopsy might be avoided. The ≤35-year threshold was chosen to explore extending the department’s existing biopsy-avoidance threshold of under 25 years for benign-appearing lesions, given the low incidence of breast cancer in this age group. A lesion was classified as eligible for biopsy avoidance when all three criteria were met: (1) patient age ≤ 35 years; (2) clinician ultrasound assessment U2 or U3; and (3) KOIOS AI assessment BI-RADS 2. All lesions not meeting all three criteria remained in the standard biopsy pathway (Figure 2).

The primary effectiveness outcome was the proportion of lesions in women aged ≤ 35 years that would have been classified for biopsy avoidance. Safety was evaluated as the number and proportion of malignant lesions classified for biopsy avoidance and the proportion of malignancies retained within the biopsy pathway. The proportion of benign lesions identified for biopsy avoidance and the benign predictive value of the no-biopsy classification were also reported.

### 2.6. Statistical Analysis

Analyses were performed at lesion level for diagnostic performance and clinical decision algorithm evaluation. Patient-level malignancy rates were also described by age group. Exact two-sided 95% confidence intervals (Clopper–Pearson) were calculated for binomial proportions used to describe the primary algorithm outcomes. The difference in patient-level malignancy prevalence between age groups was assessed using a chi-square test.

For binary diagnostic performance analyses, clinician assessments were dichotomised as U2–U3 versus U4–U5, and AI assessments as BI-RADS 1–3 versus BI-RADS 4–5, with the latter group in each system treated as test-positive for malignancy. These analytical groupings do not imply direct clinical equivalence between the classification systems or their management recommendations. Table 1 provides an illustrative correspondence between the categories. All included lesions underwent biopsy; potential biopsy avoidance was evaluated separately using the combined clinician–AI–age rule.

For conventional diagnostic performance, malignancy was treated as the positive reference outcome. Sensitivity, specificity, positive predictive value (PPV), negative predictive value (NPV) and accuracy were calculated for clinician ultrasound assessment and KOIOS AI. Agreement between clinician and AI ordinal classifications was assessed using weighted Cohen kappa with linear and quadratic weighting. Binary paired classification differences were assessed with McNemar testing. The rule-based clinical decision algorithm was implemented in Microsoft Excel version 2609, build 16.0.20430.20032 (Microsoft Corporation, Redmond, WA, USA). Statistical analyses were performed using MedCalc Statistical Software version 23.5.2 (MedCalc Software Ltd., Ostend, Belgium).

### 2.7. Exploratory Budget Impact Analysis

An illustrative budget impact analysis was undertaken from the NHS provider perspective, informed by the NICE Evidence Standards Framework for digital health technologies [18]. The model compared the current biopsy pathway with the proposed clinical decision algorithm pathway and estimated the resource consequences of avoided biopsy episodes. A five-year horizon was used, with no discounting, consistent with budget impact analysis convention. The model was implemented in HecoCost Version 2.0 (HecoAnalytics Ltd., Uxbridge, UK).

Unit costs were derived from NHS cost-collection data and local pathway costing. The principal procedure cost was ultrasound-guided biopsy (currency code YJ13Z), reported as £666 for an outpatient procedure and £853 for a day case. Histopathology and multidisciplinary review were incorporated into the pathway model, yielding an estimated difference of approximately £1000 between a patient proceeding through the biopsy pathway and one in whom biopsy was avoided. The analysis estimated gross cost offsets before any technology acquisition or commercial payment.

An illustrative value-sharing scenario was informed by indicative commercial feedback from KOIOS, under which the supplier’s payment would comprise 20% of demonstrated savings, with implementation and training included and no additional supplier charges. For this scenario, the 20% payment was applied to the modelled gross cost offsets. Additional NHS staff time associated with AI implementation and use was not quantified. Deterministic sensitivity analysis examined the principal drivers of projected savings. The model assumed discharge after the initial assessment for patients classified as potentially suitable for biopsy avoidance, with no scheduled follow-up consultations or repeat imaging. Consequently, no routine follow-up costs were included for this pathway. This assumption reflects the proposed management of lesions meeting the combined clinician–AI–age criteria as benign, consistent with discharge without routine follow-up for U2 lesions in local practice. Its clinical safety, particularly for lesions initially classified as U3, requires prospective validation. Any follow-up subsequently required would reduce the projected cost offsets.

## 3. Results

### 3.1. Cohort Characteristics

**Figure 3:**
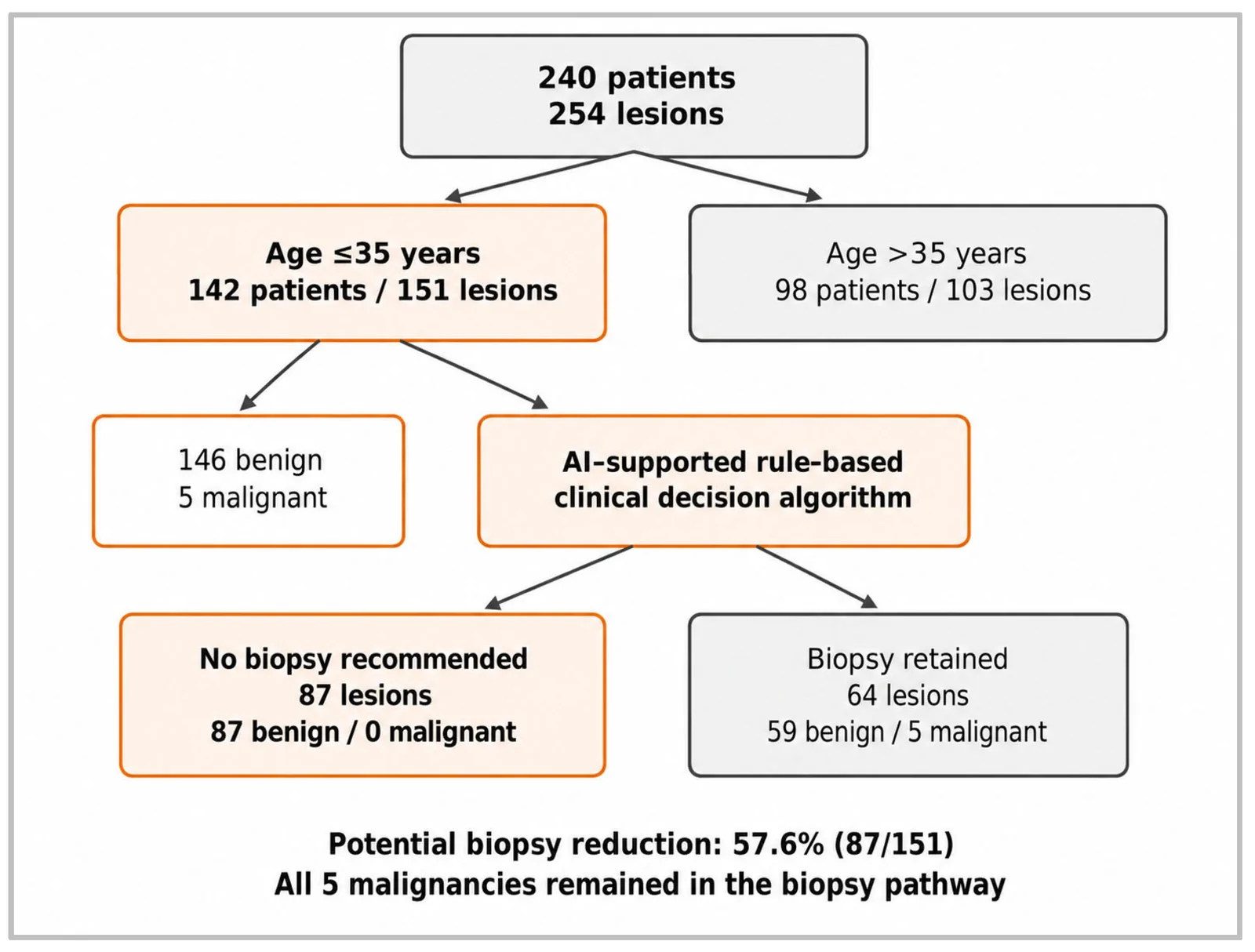
Study cohort and primary clinical decision algorithm outcome flow. The primary triage analysis was conducted within the 151 lesions from women aged 35 years or younger.

Of the 240 patients, 32 were diagnosed with cancer, yielding an overall patient-level malignancy rate of 13.3% (95% CI, 9.3–18.3%). Among patients aged ≤ 35 years, 5 of 142 (3.5%; 95% CI, 1.2–8.0%) had malignancy, compared with 27 of 98 (27.6%; 95% CI, 19.0–37.5%) in those aged > 35 years, see Figure 3. This difference was statistically significant (χ^2^ = 28.97, *p* < 0.001), demonstrating a substantially lower malignancy prevalence among patients aged ≤ 35 years and supporting the use of age as a component of risk stratification in the evaluation of breast ultrasound lesions.

### 3.2. Clinician and AI Diagnostic Performance

**Figure 4:**
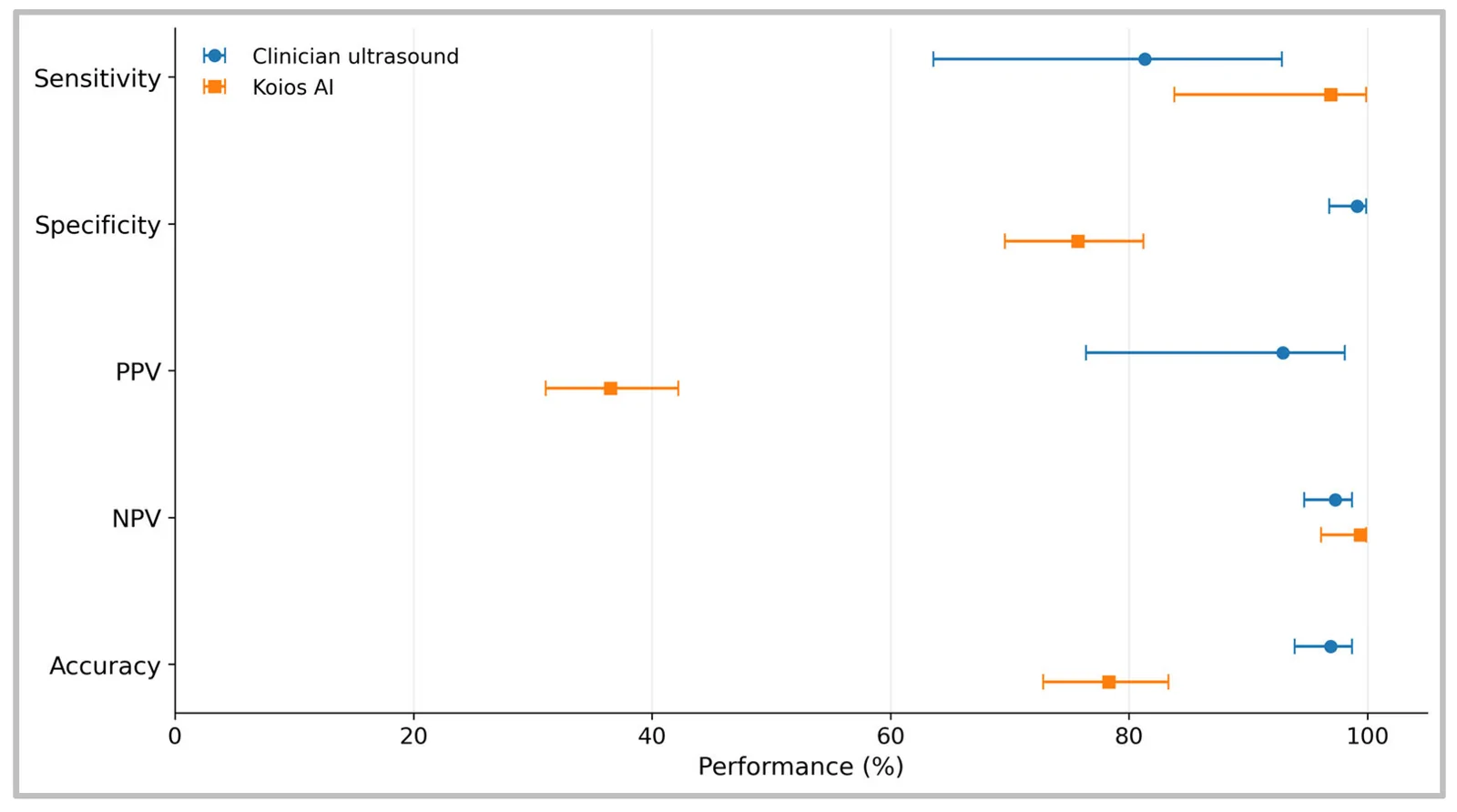
Diagnostic performance of clinician ultrasound assessment and KOIOS AI software. Points show the observed estimate and error bars show the reported 95% confidence intervals. PPV, positive predictive value; NPV, negative predictive value.

The diagnostic performance of clinician ultrasound assessment and KOIOS AI was evaluated against final benign/malignant histopathology for all 254 lesions. Clinician assessment showed high specificity (99.1%) and PPV (92.9%), whereas KOIOS AI showed higher sensitivity (96.9%) and NPV (99.4%) but lower specificity (75.7%) and PPV (36.5%) (Table 2; Figure 4). These complementary performance characteristics are consistent with use of AI as an additional rule-out support layer rather than as a standalone replacement for clinician assessment.

Weighted agreement between clinician and AI ordinal classifications was fair using linear weighting (κ = 0.27) and moderate using quadratic weighting (κ = 0.44), suggesting that many disagreements occurred between adjacent categories rather than representing extreme discordance. There were 58 lesions classified as clinician-negative/AI-positive and one classified as clinician-positive/AI-negative. AI classified a higher proportion of lesions as positive than clinician assessment (85/254 versus 28/254; paired difference, 22.44 percentage points; 95% CI, 17.20–27.69; two-sided exact McNemar test, *p* < 0.0001).

### 3.3. Clinical Decision Algorithm Performance

Overall, 87 lesions were classified as suitable for biopsy avoidance, corresponding to a biopsy avoidance rate of 57.6% (95% CI 49.3–65.6%) among lesions in patients aged 35 years or younger and 34.3% (95% CI 28.4–40.4%) across the full cohort. All 87 lesions classified for biopsy avoidance were benign, with no malignant lesions incorrectly classified as suitable for biopsy avoidance. All five malignant lesions in the ≤35-year age group remained in the biopsy pathway, corresponding to a cancer-retention rate of 100% (95% CI, 47.8–100%). Importantly, the negative predictive value was 100% (95% CI 95.8–100%) calculated as NPV = TN/(TN + FN), where TN = benign lesions correctly classified as not needing biopsy and FN = malignant lesions incorrectly classified as not needing biopsy.

Of the 87 lesions classified as potentially suitable for biopsy avoidance, 20 had a clinician U2 classification and 67 had a U3 classification. The estimated reduction in biopsy therefore predominantly involved U3 lesions with an AI BI-RADS 2 assessment in patients aged 35 years or younger. No lesions classified by clinicians as U4 or U5 qualified for biopsy avoidance under the proposed algorithm.

### 3.4. Illustrative Budget Impact

To understand the potential financial impact of deploying the clinical decision algorithm a proportionate budget impact model was constructed that costed all the potential patient journeys within the study in the breast unit. The relative proportions of patients passing through the arms of the pathway were modelled with and without the use of the clinical decision algorithm. Among the 87 biopsies that would have been avoided using the algorithm, most patients underwent only one biopsy. A small percentage were initially reported as B1, requiring additional biopsy. Furthermore, it was theoretically possible within the clinical pathway in the unit that lesions with a biopsy score of B3 could have been subsequently examined with vacuum biopsy. In this cohort no such patients were identified; it is likely, however, in a larger data set or within other institutions this may vary, with a marginal effect on estimates.

The primary driver of projected cost savings is the avoidance of the ultrasound-guided biopsy procedure (HRG/Currency Code YJ13Z), costed at £666 for an outpatient procedure and £853 for a day case, according to the NHS local cost collection data. In addition, RD40Z for an ultrasound scan with duration of less than 20 min, without contrast, is priced at £61. Including histopathological processing and multidisciplinary team review, the estimated total cost of the biopsy pathway was approximately £1278. Within the limits of estimation, the incremental cost compared with assessment and discharge without biopsy was approximately £1000, with variation arising from outpatient or day-case delivery, the applicable HRG costs and the costing of other pathway components. Within this patient cohort over the period of the evaluation alone implementation of the clinical decision algorithm would have saved approximately £85,000.

Approximately 10,000 patients attend the LTHT Breast Unit annually [15]. Local activity data indicated that 37.8% are aged ≤ 35 years, with approximately 1.1 lesions per patient and around 400 biopsies annually in this age group. Applying the observed 57.6% biopsy-avoidance proportion to this activity generated a five-year gross budget impact estimate of approximately £1.16 million under full implementation. Under this illustrative arrangement, the projected cost offset after the supplier payment would be approximately £0.93 million over five years, before allowing for any additional NHS staff time associated with AI implementation and use. Deterministic sensitivity analysis identified the cost of avoided biopsy episodes, the proportion of attendees aged ≤ 35 years and the biopsy-avoidance rate as the principal drivers of the result.

## 4. Discussion

### 4.1. Principal Findings

This service evaluation demonstrates that a simple rule-based strategy combining age, clinician ultrasound assessment and AI decision support could materially reduce benign biopsy activity in younger women. Among lesions in women aged ≤ 35 years, 57.6% would have been assigned to biopsy avoidance, and all 87 lesions in that group were benign on histopathology. All five malignancies in the target age group remained in the biopsy pathway. The result is clinically important because it addresses the practical question of whether AI can support a change in pathway, rather than merely whether an AI system can reproduce radiological classification.

The clinician and AI assessments showed different performance characteristics. Clinician assessment was highly specific, whereas KOIOS AI was more sensitive but generated more false-positive classifications. The differing thresholds may partly explain this pattern: classifying U3 as test-negative favours clinician specificity, while classifying BI-RADS 4A as test-positive favours AI sensitivity. These results describe performance at the chosen thresholds and should not be interpreted as an intrinsic advantage of either method. The modest agreement between the two assessments suggests that they were not simply duplicating the same information. The proposed clinical decision algorithm retains clinician assessment as an essential component, combining it with AI output and age to identify lesions potentially suitable for biopsy avoidance. This concept is consistent with broader decision-referral approaches in breast imaging, in which the value of AI derives from interaction with the clinician rather than autonomous diagnosis [7,8,9,10].

### 4.2. Clinical Interpretation and Safety

The absence of malignancy among the 87 lesions assigned to no biopsy is encouraging, however, the 95% confidence interval for the observed 0/87 malignancy rate extends to 4.2%, and only five malignancies occurred in the ≤35-year target population. The corresponding 95% confidence interval around the observed 5/5 cancer-retention rate is therefore wide (47.8–100%). A larger prospective cohort is required to estimate the missed-cancer risk with the precision needed for clinical implementation.

This distinction is important because the proposed algorithm is a triage rule, not a replacement for histopathology where biopsy remains indicated. It is also intended for a defined low-risk subgroup rather than for lesions with suspicious clinical or imaging features. Clinical history, including personal and family history of breast or ovarian cancer, known genetic predisposition and changes such as lesion growth, should inform decisions about biopsy avoidance. A future biopsy-avoidance pathway should exclude patients with suspicious axillary findings. Incorporating these additional factors alongside age, clinician ultrasound assessment and AI classification may further refine patient selection and should be evaluated in a prospective multicentre study. U3 lesions normally undergo biopsy in UK practice, and RCR guidance permitting biopsy avoidance in younger women applies to lesions with typical fibroadenoma features rather than indeterminate U3 lesions [3,5,6]. The proposed algorithm explores whether combining a clinician U2 or U3 assessment with KOIOS BI-RADS 2 in women aged ≤ 35 years can support a benign overall assessment and biopsy avoidance. Although all 67 U3 lesions meeting these criteria were benign on histopathology, all underwent biopsy; the safety of withholding biopsy was therefore not tested. In a future trial, interval cancers could be identified through scheduled clinical and ultrasound follow-up, prompt reassessment of new or changing symptoms, and linkage to hospital and cancer-registration records to capture diagnoses made outside the participating breast units. The follow-up intervals and duration would be prespecified in the trial protocol. The implementation of these findings would need to be evaluated in a prospective multicentre trial with appropriate follow up to capture interval cancers.

### 4.3. Economic and Service Implications

Avoided biopsy has potential benefits beyond direct procedure cost. Breast biopsy can cause discomfort, anxiety and procedure-related complications, and it generates pathology and multidisciplinary review workload [19,20]. Reducing benign biopsies could therefore release diagnostic capacity at a time of sustained pressure on breast services and increasing referral demand [1,2]. The illustrative budget model estimated approximately £85,000 in gross cost offsets within the evaluation cohort and £1.16 million over five years under full implementation at LTHT.

These estimates should be interpreted as provider-level resource consequences rather than automatically cash-releasing savings. Many NHS costs are fixed in the short term, and lower biopsy activity may primarily release capacity rather than reduce expenditure. The distinction between gross cost offsets, cash savings and capacity benefits should therefore be explicit in any procurement or value-sharing arrangement. A performance-linked commercial model may align payment with realised benefit, but it also requires agreed baselines, attribution rules, audit mechanisms and clarity over which organisation bears technology cost and which captures the benefit.

### 4.4. Comparison with Previous Evidence

Previous studies of KOIOS and other AI systems in breast ultrasound have mainly focused on diagnostic performance or changes in BI-RADS assessment [9,10,11,12]. Browne et al. reported the potential for AI to improve the predictive value of ultrasound biopsy decisions, while Mango et al. evaluated the effect of AI decision support on lesion assessment [11,12]. The present evaluation adds to this literature by embedding AI within an explicit age- and clinician-dependent pathway rule and by quantifying the associated biopsy and resource consequences in an NHS setting. The purpose is therefore not to establish superiority of KOIOS over clinicians but to evaluate whether complementary human and AI assessments can support safer de-escalation of an invasive diagnostic procedure.

### 4.5. Limitations

This study had several recognised limitations. First, this was a single-centre service evaluation with a local case mix, imaging practice, reader experience and biopsy thresholds that may differ across institutions. Second, although patients were included consecutively, the analysis was restricted to biopsied lesions; prevalence-dependent measures such as PPV and NPV should therefore not be extrapolated directly to all symptomatic breast-clinic populations. Third, only five malignancies occurred in the ≤35-year target group, producing uncertainty around the missed-cancer risk despite no observed malignant no-biopsy classifications.

The analysis was performed at lesion level and included bilateral lesions from some patients, meaning that observations were not fully independent. The sample size was insufficient for a patient-clustered or multilevel analysis. Individual ultrasound reader identity was not recorded, preventing analysis of reader-level variability or interaction between AI and reader experience.

Clinician assessments were based on real-time ultrasound in the clinical setting, whereas KOIOS assessed selected static images. Differences in the information available to each method, together with the potential influence of image selection, should be considered when interpreting their comparative diagnostic performance.

Finally, the economic analysis was illustrative and based on modelled rather than prospectively realised savings. Cost structures, the proportion of outpatient versus day-case biopsies, pathology costs, technology price and the extent to which released capacity can be converted into cash savings will vary between providers. Additional NHS staff time associated with AI implementation and use was not quantified. A future prospective multicentre trial should measure staff time and actual implementation and follow-up costs to provide a complete assessment of the net budget impact, alongside evaluation of diagnostic safety, patient-reported outcomes and operational effects.

## 5. Conclusions

In this retrospective analysis of an NHS service evaluation, an AI-supported rule-based clinical decision algorithm identified 87 of 151 lesions in women aged 35 years or younger as potentially suitable for biopsy avoidance, representing a 57.6% reduction in biopsy activity within the target subgroup. Of these, 67 (77.0%) had a clinician U3 classification. All 87 lesions were benign and all five malignancies remained in the biopsy pathway. These findings support the concept that AI may add value as a safety layer within a clinician-led, age-stratified decision rule rather than as a standalone diagnostic replacement. Because the number of malignancies was small and the study was conducted at a single centre using a biopsy cohort, prospective multicentre validation with explicit safety thresholds and real-world resource measurement is required before routine implementation.

## Figures and Tables

**Table 1:** Illustrative correspondence between UK breast imaging categories and BI-RADS categories, based on Taylor et al. [17].

UK Breast Ultrasound Assessment Category	Description	BI-RADS
U1	Normal/no significant abnormality	1 (negative)
U2	Benign	2—Benign, or 3—Probably benign, depending on lesion characteristics
U3	Indeterminate/probably benign	4a (suspicious–low) 4b (suspicious–medium)
U4	Findings suspicious of malignancy	4c (suspicious–high)
U5	highly suspicious for malignancy	5 (highly suggestive of malignancy)

**Table 2:** Lesion-level diagnostic performance of clinician ultrasound assessment and KOIOS AI software against final histopathology (*n* = 254 lesions).

Metric	Clinician Ultrasound (95% CI)	KOIOS AI (95% CI)
Sensitivity	81.3% (63.6–92.8%)	96.9% (83.8–99.9%)
Specificity	99.1% (96.8–99.9%)	75.7% (69.5–81.2%)
Positive predictive value	92.9% (76.4–98.1%)	36.5% (31.1–42.2%)
Negative predictive value	97.3% (94.7–98.7%)	99.4% (96.1–99.9%)
Accuracy	96.9% (93.9–98.6%)	78.3% (72.8–83.3%)

## Data Availability

The data that support the findings of this study are available from the corresponding author upon reasonable request, subject to institutional approvals and applicable patient-confidentiality requirements.

## Abbreviations

The following abbreviations are used in this manuscript: AI, artificial intelligence; AUC, area under the curve; BIA, budget impact analysis; BI-RADS, Breast Imaging Reporting and Data System; CI, confidence interval; DS, decision support; HRG, Healthcare Resource Group; LTHT, Leeds Teaching Hospitals NHS Trust; MDM, multidisciplinary meeting; NHS, National Health Service; NICE, National Institute for Health and Care Excellence; NPV, negative predictive value; PACS, Picture Archiving and Communication System; PLICS, Patient-Level Information and Costing System; PPV, positive predictive value.
